# Effect of Pentoxifylline on Staurosporine-Induced Neurite Elongation in PC12 Cells

**DOI:** 10.31557/APJCP.2019.20.9.2633

**Published:** 2019

**Authors:** Loghman Diojan, Hossein Zhaleh, Mehri Azadbakht, Ali Bidmeshkipour, Ehsan Khodamoradi

**Affiliations:** 1 *Department of Radiology and Nuclear Medicine, School of Allied Medical Sciences, *; 2 *Substance Abuse Prevention Research Center, Institute of Health, Kermanshah University of Medical Sciences,*; 3 *Department of Biology, Faculty of Sciences, Razi University, Kermanshah, Iran.*

**Keywords:** Pentoxifylline, cell death, Neurite, Staurosporine

## Abstract

**Objective:**

Pentoxifylline enhances neurite elongation in PC12 cells. This study investigated the effects of pentoxifylline on staurosporine-induced neurite elongation in PC12 cells.

**Materials and Methods:**

There were five treatment groups, including treatment group I (1 nM), treatment group II (10 nM), treatment group III (100 nM), treatment group IV (1uM), and treatment group V (10 mM of pentoxifylline), together with 214 nM staurosporine for a range of time (6, 12 and 24 hours). Cells only treated with staurosporine at a concentration of 214 nM were used as the control group. Cell proliferation, cell death, immunocytochemistry assay, and Total Neurite Length were assessed.

**Results:**

The results showed that pentoxifylline increased cell viability (p<0.05) in a dose- and time-dependent manner, and cell death assay showed that cell death decreased in a dose- and time-dependent manner (p<0.05). TNL increased significantly compared with control cells (p<0.05). Immunocytochemistry assay showed that pentoxifylline at low and high concentrations enhanced β-tubulin III and GFAP protein expression compared with control cells.

**Conclusion:**

It can be concluded that pentoxifylline has positive effects on the staurosporine-induced neurite outgrowth process in PC12 cells.

## Introduction

Staurosporine is a biological matter that induces neurite outgrowthat low concentrations (nM) and apoptosis at high concentrations (µM) in several cells (Schumacher et al., 2003; Das et al., 2004; Giuliano et al., 2004; Faghihi et al., 2008). Staurosporine inhibits some protein kinases such as protein kinase C (PKC), a family of serine/threonine kinases (Senderowicz, 2005) which may contribute to neurite outgrowth, cell proliferation, and cell differentiation (Jin et al., 2015). Pentoxifylline can inhibit the phosphodiesterase (PDE) enzyme activity, leading to an increase in cyclic adenosine monophosphate (cAMP) (Joshi et al., 2014). Previous studies have demonstrated that pentoxifylline may exert its effects through several mechanisms including translocation of extracellular calcium, increased cAMP and cyclic guanosine monophosphate (cGMP) caused by inhibition of phosphodiesterase, and blockade of adenosine receptors (Nasiri-Toosi et al., 2013; Speer et al., 2017) and cAMP overload pathway, resulting in cell viability and cell proliferation in neuronal cells (Cui and So, 2004; Hannila and Filbin, 2008).

An increasing number of reports have shown that PTX has many anti-inflammatory/immunomodulatory activities by reducing the production of several cytokines. Moreover, PTX and other PDE inhibitors exert their anti-inflammatory/immunomodulatory activities by interfering with production of many cytokines (IL-4, IL-5, IL-10, TNF-α, IL-2 etc.) through inhibition of NF-kB and NFAT and stimulation of AP-1 and CREBs (cAMP response element binding proteins)(Hannila and Filbin, 2008). Furthermore, the effect of pentoxifylline on cytokines production seems to be due, at least in part, to an increase in intracellular cAMP levels in inflammatory cells. The results of a study by Sirin et al., (1998) showed that pentoxifylline reduces cerebral injury and preserves the neurologic function in transient global ischemia in rats.

The present study was designed to investigate the effects of different concentrations of pentoxifylline on staurosporine-induced neurite elongation, cell viability, and cell death in bone marrow mesenchymal stem cells. 

## Materials and Methods

*Cell lines*

In this experimental study, PC12 cells were cultured in the RPMI1640 culture medium (Gibco), supplemented with 5% FBS (Gibco), 100 u/ml penicillin (Sigma), and 100 mg/ml streptomycin (Sigma). The cells were incubated at 37 °C in a humidified environment containing 5% CO_2_.

**Figure 1:**
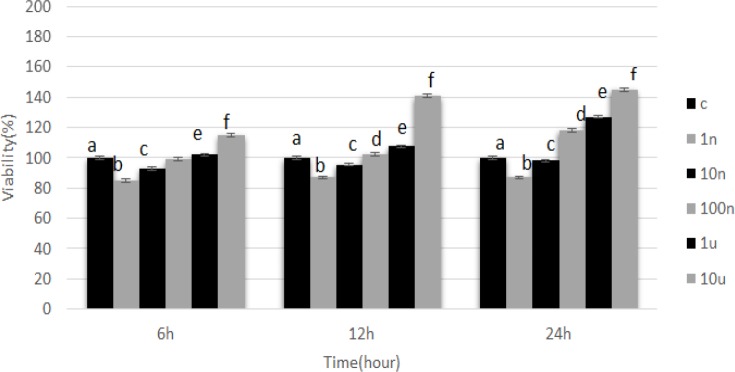
Effect of Pentoxifylline in the Presence of Staurosporine on Viability of PC12 Cells. Cells were treated with different concentrations of pentoxifylline (I: 1 nM, II: 10 nM, III: 100 nM, IV: 1 uM, V: 10 uM) and then treated with 214 nM staurosporine. Cells only treated with 214 nM staurosporine were used as the control group. ^a,b,c,d^ p < 0.05; A significant difference was seen between the experiment and control groups

**Figure 2:**
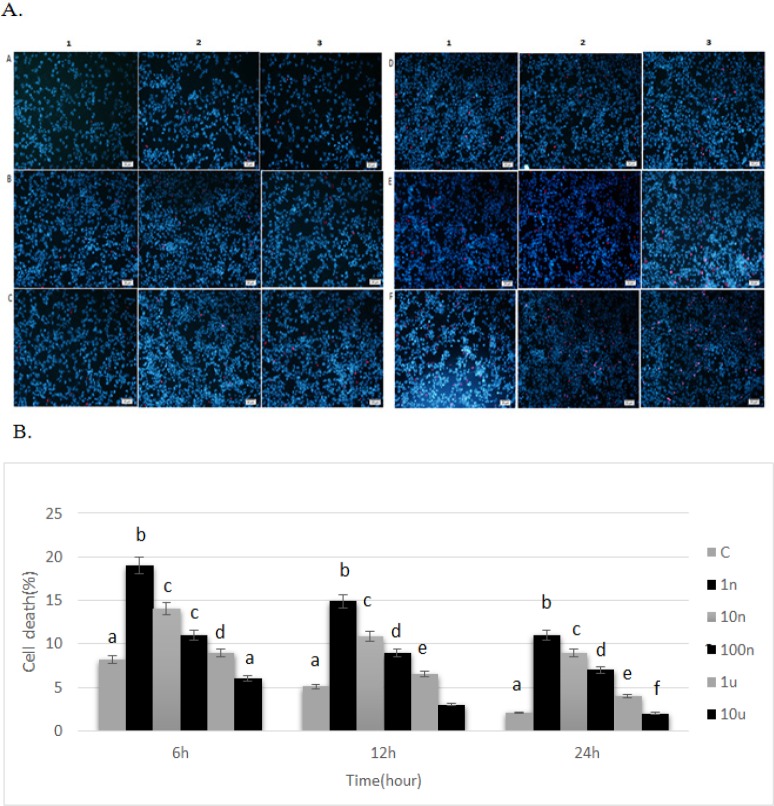
A. Effect of pentoxifylline in the presence of staurosporine on cell death of PC12 cells after 6 hours (column 1), 12 hours (column 2), and 24 hours (column 3). A= Control, B=treatment group 5, C=treatment group 4, D=treatment group 3, E=treatment group 2, F=treatment group 1. B. Effect of pentoxifylline in the presence of staurosporine on cell death of PC12 cells. Cells were treated with different concentrations of pentoxifylline (I: 1 nM, II: 10 nM, III: 100 nM, IV: 1 uM, V: 10 uM) and then treated with 214 nM staurosporine. Cells only treated with 214 nM staurosporine were used as the control group. ^a,b,c,d^ p < 0.05; a significant difference was seen between the experiment and control groups

**Figure 3:**
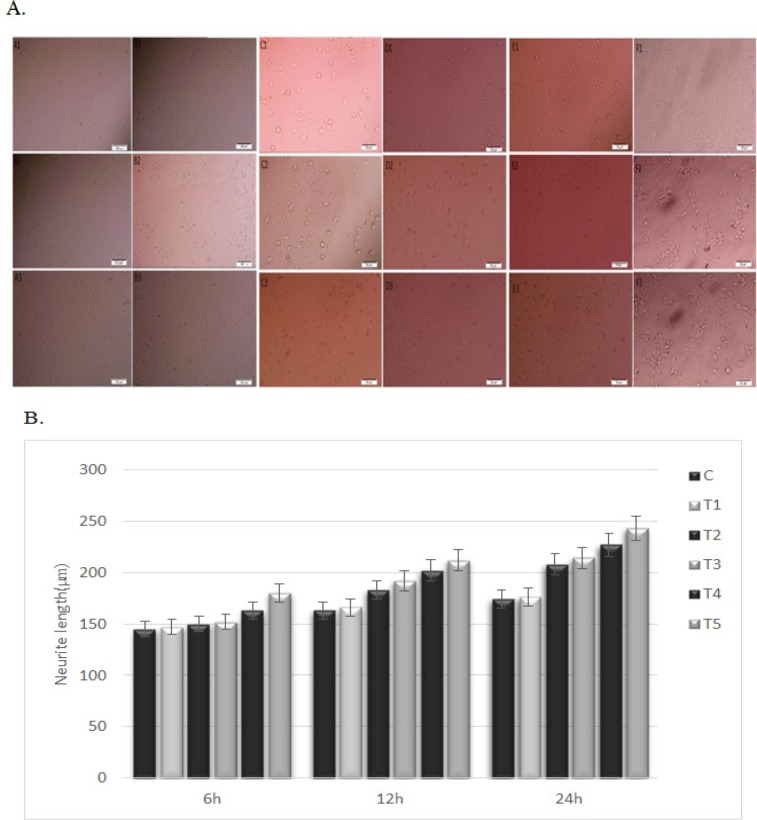
A. Effect of pentoxifylline in the presence of staurosporine on the total neurite length of PC12 cells after 6 hours (A1-F1), 12 hours (A2-F2), and 24 hours (A3-F3). A1-A3= Control, B1-B3=treatment group 1, C1-C3=treatment group 2, D1-D3=treatment group 3, E1-E3=treatment group 4, F1-F3=treatment group 5. B. Effect of pentoxifylline in the presence of staurosporine on total neurite length of PC12 cells. Cells were treated with different concentrations of pentoxifylline (I: 1 nM, II: 10 nM, III: 100 nM, IV: 1 uM, V: 10 uM) and then treated with 214 nM staurosporine. Cells only treated with 214 nM staurosporine were used as the control group. a,b,c,d p < 0.05; a significant difference was seen between the experiment and control groups

**Figure 4:**
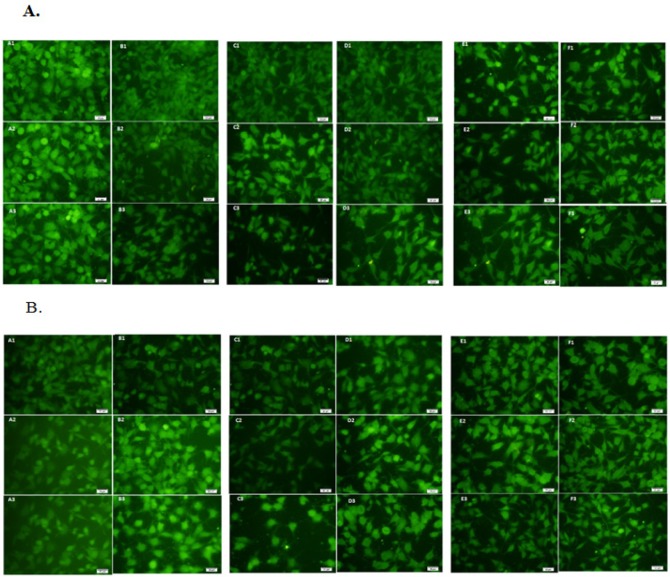
A. Immunocytochemistry assay of β-tubulin III in PC12 cells after 6 hours (A1-F1), 12 hours (A2-F2), and 24 hours (A3-F3); A1-A3, Control; B1-B3, treatment group 1; C1-C3, treatment group 2; D1-D3, treatment group 3; E1-E3, treatment group 4; F1-F3, treatment group 5; B. Immunocytochemistry assay of GFAP in PC12 cells after 6 hours (A1-F1), 12 hours (A2-F2), and 24 hours (A3-F3); A1-A3, Control; B1-B3, treatment group 1; C1-C3, treatment group 2; D1-D3, treatment group 3; E1-E3, treatment group 4; F1-F3, treatment group 5

**Figure 5:**
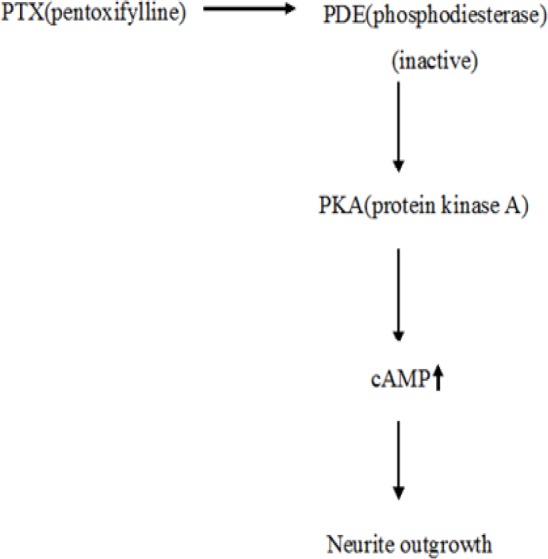
Scheme of PDE Inactivation by Pentoxifylline through a Protein Kinase-Dependent Mechanism (PKC, PKA), Leading to Increased cAMP, Neurite Elongation, and Neuroglial Protein Marker Expression in the PC12 Cell Line

*Cell treatment*

PC12 cells were plated overnight, washed with PBS (pH=7.4), and were then cultured. Cells were treated with pentoxifylline at different concentrations (group I: 1 nM, group II: 10 nM, group III: 100 nM, group IV: 1 uM, group V: 10 uM,), and then treated with 214 nM staurosporine as a neurite outgrowth inducer (for 6, 12 and 24 hours). Cells only treated with staurosporine at a concentration of 214 nM were used as control cells. The cells were incubated at 37°C with 5% CO_2_.

*Cell proliferation measurement*

The percentage of cell proliferation was measured by the MTT assay (Khatibi et al., 2017). The cells were cultured in a 96-well plate at a density of 1×10^4^ cell/well in the RPMI1640 culture medium containing 0.2% BSA added overnight. Then, the cells were cultured with different treatment media as described (for 6, 12, and 24 hours). The optical density of each well was measured using a microplate reader (EL800; USA). 

*Quantification of cell death*

The cell death index was calculated as described previously (Yamasaki, 2003). Briefly, the cells were cultured in 96-well culture plates at a density of 5×10^3 ^cells/well overnight. The cells were treated in different treatment media for different periods (6, 12 and 24 hours). Then, the cells were incubated at 37°C for 30 minutes using the Hoechst 33,342 dye (10 mg/ml in PBS) and washed twice in PBS. PI (50 mg/ml in PBS) was added just before microscopy. The cells were visualized using an inverted florescence microscope (Olympus IX-71, Japan). The apoptotic index was then calculated. 

*Measurement of total neurite length (TNL)*

The TNL was measured as reported in a previous study (Rønn et al., 2000). The cells were plated in 24-well culture plates at a density of 6×10^4^ cells/well overnight. The cells were treated in different treatment media for certain periods of time (6, 12 and 24 hours) and fixed. The morphology of the cells was assessed by an inverted microscope (Olympus IX-71, Japan). 

*Immunocytochemistry*

The cells were plated in 96-well culture plates at a density of 1×10^4^ cells/well overnight. Then, the cells were treated in different treatment media for 6 hours, fixed, and permeabilized in 0.1% Triton/PBS for 5 minutes. The cells were incubated in PBS containing 0.5% bovine serum albumin and 0.1% Tween 20 for 30 minutes to reduce nonspecific binding, followed by overnight incubation at 4°C with the following rabbit polyclonal Abs: β-tubulin III (1:40; Sigma) and GFAP (1:80; Sigma). After washing, FITC-conjugated (green) secondary Ab (1:100; Santa Cruz Biotechnology) was applied for 1 h at room temperature. The wells were treated with an anti-fade reagent (Molecular Probes, Inc.) and examined for immunofluorescence under a fluorescent microscope (Olympus AX-70).

*Statistical analysis*

Data are expressed as mean ± SEM. All calculations were performed by SPSS (version 19; SPSS Inc.). The differences in the percentage of viability, apoptotic index, and total neurite length between different treatment groups were analyzed using t-test at a significance level of 0.05.

## Results

*Cell proliferation*

The percentage of cell proliferation in cells cultured in a culture medium containing pentoxifylline and staurosporine was assessed by the MTT assay. After 6 hours, cell proliferation assay showed that cell proliferation decreased in treatment groups 1 and 2 compared with control group (p<0.05). In treatment group 5, cell proliferation increased when compared with the control group, but in treatment groups 3 and 4, differences in cell proliferation were not significant (p<0.05). The lowest percentage of cell proliferation after 6 hours was seen in treatment group 1 (85%) and the highest was observed in treatment group 5 (115%) (p<0.05).

After 12 hours, cell proliferation assay showed that cell proliferation decreased in treatment groups 1 and 2 compared with the control group (p<0.05). In treatment groups 3-5, cell proliferation increased significantly as compared to the control group (p<0.05). The lowest and highest percentage of cell proliferation after 12 hours was seen in treatment group 1 (87%) and treatment group 5 (140%), respectively (p<0.05).

After 24 hours, cell proliferation assay showed that cell proliferation decreased in treatment groups 1 and 2 compared with control group (p<0.05). In treatment groups 3-5, cell proliferation increased significantly as compared with the control group (p<0.05). The lowest and highest percentage of cell proliferation after 24 hours was seen in treatment group 1 (87%) and treatment group 5 (145%), respectively (p<0.05).

*Cell death*

The apoptotic effect of pentoxifylline on PC12 cells cultured in culture media containing different concentrations of pentoxifylline and staurosporine was assessed by PI/Hoechst florescence staining (Figure 2A). Hoechst/PI staining assay showed that with an increase in the concentration of pntoxifylline, cell death decreased over time (p<0.05). 

After 6 hours, the percentage of cell death in treatment groups 1-3 increased as compared with the control group and the percentage of cell death in treatment groups 4 and 5 was similar to the control group. The highest percentage of cell death was seen in treatment group 1 (11%) and the lowest percentage of cell death was observed in treatment group 5 (2.8%). 

After 12 hours, the percentage of cell death in treatment groups 1-3 increased when compared to the control group but the percentage of cell death in treatment groups 4 and 5 was similar to the control group (p<0.05). The highest and lowest percentage of cell death was seen in treatment group 1 (14.9%) and treatment group 5 (3.8%), respectively. 

After 24 hours, the percentage of cell death in treatment groups 1-3 increased when compared to control group while the percentage of cell death in treatment groups 4 and 5 was similar to the control group (p<0.05). The highest and lowest percentage of cell death was seen in treatment group 1 (19%) and treatment group 5 (6.9%), respectively (Figure 2A). 

*Neurite outgrowth measurement*

The mean TNL for PC12 cells was assessed using an inverted microscope (Figure 3A). The total neurite length was calculated, which showed that pentoxifylline concentrations enhanced neurite outgrowth (p<0.05) (Figure 3A). After 6 hours, TNL increased in treatment groups 2-5 when compared to the control group. TNL also increased with an increase in the concentration of pentoxifylline (p<0.05). TNL in treatment group 1 was similar to TNL in the control group. The highest and lowest TNL was seen in treatment group 5 and the control group, respectively (p<0.05). 

After 12 hours, TNL increased in treatment groups 2-5 when compared to the control group. TNL also increased with an increase in the concentration of pentoxifylline (p<0.05). TNL in treatment group 1 was similar to TNL in the control group. The highest and lowest TNL was seen in treatment group 5 and the control group, respectively (p<0.05).

After 24 hours, TNL increased in treatment groups 2-5 as compared to the control group. TNL also increased with an increase in the concentration of pentoxifylline over time (p<0.05). TNL in treatment group 1 was similar to TNL in the control group. The highest and lowest TNL was seen in treatment group 5 and the control group, respectively (p<0.05).

*Immunocytochemistry assay*

The effect of pentoxifylline in the presence of staurosporine on PC12 cells was characterized by immunocytochemistry study after 24 hours exposure. For immunocytochemistry study, cultured cells were stained with β-tubulin III and GFAP as neurological biomarker antibodies. The showed that pentoxifylline at different concentrations (in different treatment media) enhanced PC12 cells β-tubulin III (Figure 4A) and GFAP (Figure 4B) as compared to the control group. 

## Discussion

In the current study, we investigated the effect of pentoxifylline on staurosporine induced neurite outgrowth in PC12 cells. It has been shown that staurosporine, as a fungal chemical matter, can induce neuronal differentiation in neuronal cells (Frassetto et al., 2006). It can induce neuronal differentiation by neurite outgrowth in PC12 cells (Rasouly et al., 1996; Das et al., 2004). In the present investigation, it was used as a biological inducer of neurite elongation. Staurosporine is a potent inhibitor of a number of kinases like PKC, PKA, and tyrosine protein kinase (Gani and Engh, 2010). In the present study, we used the pentoxifylline to enhance neurite elongation in PC12 cells. Pentoxifylline as a phosphodiesterase enzyme inhibitor increases the concentrations of intracellular cAMP. Many studies have demonstrated that increased cAMP enhances the growth and survival of the neurons. It has been shown that the cells differentiate into early neural progenitors under conditions that increase intracellular cAMP. Following a treatment that elevates intracellular levels of cAMP, about 25% of hMSCs assume a neuron-like morphology (Deng et al., 2001). It has been already reported that cAMP induces mechanisms for maturation of neuronal progenitor cells (Lepski et al., 2013). The results of our study showed when cells are treated with pentoxifylline and staurosporine, cell proliferation (as shown in Figure 1) and neurite outgrowth (as shown in Figure 3) increase and cell death (as shown in Figure 2) decreases (p<0.05). It is possible that an increase in the cAMP concentration induces cell proliferation and neurite elongation and suppresses cell death in treatment groups more than the control group. Pentoxifylline regulates the neurite outgrowth process as well as GFAP and β-tubulin III protein expression through activation of the PKA pathway and capacitative Ca^2+^ influx. Development, neuronal survival, and differentiation can be influenced by a variety of local signals or signals derived from intermediate or final target tissues. therefore, in this study, we suggest it is possible that pentoxifylline enhances neurite elongation and neurological protein marker expression in PC12 treated by staurosporine through a protein kinase-dependent mechanism (PKC, PKA) and an increase in cAMP.

In conclusion, according to the results of the present study, pentoxifylline, possibly through increasing the cAMP level, may enhance neurite elongation and increase protein expression. However, more key factors need to be investigated in these effects.
